# Beyond Medical Care: How Different National Models of Care Impact the Experience of Adolescent and Young Adult Cancer Patients

**DOI:** 10.1089/jayao.2022.0182

**Published:** 2023-12-13

**Authors:** Pandora Patterson, Rebecca L. Jacobsen, Fiona E.J. McDonald, Chaya M. Pflugeisen, Kit Bibby, Catherine Fiona Macpherson, Kate Thompson, Andrew Murnane, Antoinette Anazodo, Ursula M. Sansom-Daly, Michael P. Osborn, Allan Hayward, Cindy Kok, Rebecca H. Johnson

**Affiliations:** ^1^ Impact and Patient Programs, Canteen Australia, Sydney, Australia.; ^2^ Faculty of Medicine and Health, University of Sydney, Sydney, Australia.; ^3^ Mary Bridge Children's Hospital, MultiCare Health System, Tacoma, Washington, USA.; ^4^ Institute for Research and Innovation, MultiCare Health System, Tacoma, Washington, USA.; ^5^ Seattle Children's Hospital, Seattle, Washington, USA.; ^6^ ONTrac at Peter Mac Victorian Adolescent and Young Adult Cancer Service, Melbourne, Australia.; ^7^ School of Health Sciences, Faculty of Medicine, Dentistry and Health Sciences, University of Melbourne, Melbourne, Australia.; ^8^ NSW/ACT Youth Cancer Service, Sydney, Australia.; ^9^ Behavioural Sciences Unit, Kids Cancer Centre, Sydney Children's Hospital, Randwick, Australia.; ^10^ Discipline of Paediatrics, School of Clinical Medicine, UNSW Medicine and Health, Randwick Clinical Campus, University of New South Wales Sydney, Sydney, Australia.; ^11^ SA/NT Youth Cancer Service, Adelaide, Australia.; ^12^ Haematology/Oncology, Women's and Children's Hospital, North Adelaide, Australia.; ^13^ Adelaide Medical School, University of Adelaide, Adelaide, Australia.

**Keywords:** experience of care, PREMs, adolescent and young adult cancer, satisfaction, international study, patient experiences

## Abstract

Patient experience is positively associated with clinical effectiveness, quality care, and patient safety. This study examines the experience of care of adolescents and young adult (AYA) cancer patients from Australia and the United States, allowing a comparison of patient experiences in the context of different national models of cancer care delivery. Participants (*n* = 190) were aged 15–29 years and received cancer treatment from 2014 to 2019. Australians (*n* = 118) were recruited nationally by health care professionals. U.S. participants (*n* = 72) were recruited nationally via social media. The survey included demographic and disease variables, and questions regarding medical treatment, information and support provision, care coordination, and satisfaction across the treatment pathway. Sensitivity analyses examined the possible contribution of age and gender. Most patients from both countries were satisfied or very satisfied with their medical treatment (chemotherapy, radiotherapy, and surgery). There were significant differences between countries in the provision of fertility preservation services, age-appropriate communication, and psychosocial support. Our findings suggest when a national system of oversight with both state and federal funding is implemented, as is the case in Australia but not in the United States, significantly more AYAs with cancer receive age-appropriate information and support services, and improved access to specialist services such as fertility care. A national approach with government funding and centralized accountability appears to be associated with substantial benefits for the well-being of AYAs undergoing cancer treatment.

## Background

The global burden of cancer is significant among adolescents and young adults (AYAs).^1^ While the incidence is lower among AYAs than older adults,^2^ and the 5-year relative survival rate is higher,^3,4^ the aftermath of cancer can reverberate across decades of survivorship.^5,6^ Age-appropriate care is critically important for AYAs with cancer and can improve medical and psychosocial outcomes.^7–10^

A cancer diagnosis during the AYA period coincides with developmentally impactful life-stages. It can profoundly impact identity formation, transition to independence, peer and romantic relationships, education, and employment.^11^ AYAs with cancer experience inferior psychosocial outcomes compared with their child and adult counterparts,^12^ underscoring the need for developmentally relevant support. However, neither pediatric nor adult health care settings are fully equipped to meet their needs. In response, new models of care have emerged internationally to provide specialized support, including age-appropriate information and communication, access to fertility and sexuality counseling, and psychosocial support.^7–9,13,14^

In Australia, most AYAs receive cancer care through public hospitals, within a universal tax-funded health care system.^15^ The five Youth Cancer Services (YCS) based in major hospitals are funded by federal and state governments, with co-investment and management by Canteen, a national charity. They care for about 75% of AYAs (defined as 15–25 years) who require hospital-based treatment for their cancer.^16^

The YCS comprise five lead sites in major hospitals, which work with ∼30 other hospitals providing a national network of care.^15^ They are guided by the Australian Youth Cancer Framework^17^ that outlines key components of quality care, including national coordination of cancer services, increased access to support services and clinical trials, comprehensive medical and psychosocial assessment, and multidisciplinary expertise in AYA care.^17,18^

By comparison, in the United States, most AYAs (defined as 15–39 years) are treated in community hospital cancer clinics or academic medical centers. Treatment costs are covered by the patient's or their parent's insurance. AYAs only comprise 10% of patients within any clinic^19^; however, 90,000 are diagnosed annually. A minority of treatment facilities offer specific services for AYAs, and there is no coordination of services between hospital systems. There are currently no nationally funded government support programs to provide oversight for AYA care in the United States.

While health care delivery success has traditionally been measured using clinical outcomes, there has been increasing emphasis on patient-reported outcomes as indicators of quality of care.^20^ Patient-reported experience measures (PREMs), which report the alignment between patient experience and best practice recommendations for cancer care,^21^ can provide valuable information to inform improvements in health care practice and are increasingly being collected for AYAs, including in the UK, North America, and Australia.^22^

We collected PREMs data from AYAs with cancer in the United States and Australia, allowing a comparison of patient experiences between these two countries. We focused on the following generally recognized priority areas for AYA-specific cancer care: fertility provision, age-appropriate communication, and psychosocial support^23^ as well as overall satisfaction with cancer treatment and service. Our aim was to examine how differences in patients' experiences can be understood in the context of their respective countries' models of AYA cancer care delivery and oversight.

## Methods

### Study design and setting

This cross-sectional descriptive study used a self-report survey to investigate the experiences of care of AYAs across Australia and the United States. The study was approved for Australia by Sydney Children's Hospital Ethics Committee (Ref.: 2019/11960) and for the United States by MultiCare Health System, Tacoma, Washington (Ref.: 2017/05/4). Informed consent was implied by survey completion.

### Participants and recruitment

In Australia, the survey was distributed through the YCS. Most participants came from Victoria and New South Wales, the two most populous states. Patients aged 15–25 years at diagnosis, who completed active treatment between 2015 and 2019, were invited by health care professionals to complete an online survey via SurveyMonkey.

The U.S. participants were AYAs aged 15–39 at diagnosis and primarily recruited via social media. They received their care in many academic and community cancer centers spread over a broad geographic area comprising numerous U.S. states.^24^ Surveys were completed online via REDCap. To better align with the Australian dataset, this comparison used the subset of the U.S. respondents diagnosed between 2014 and 2018 while aged 15–29 years.

### Measures

#### Experience of care survey

The young person's experience of care survey (YPEoC) was adapted from the AYA Experience of Care survey developed by Cancer Council Victoria.^22^ It was piloted with Canteen's National Youth Advisory Group to assess its relevance, clarity, and appropriateness.

The Australian version contained a maximum of 117 questions and covered the following domains: finding out you had cancer; deciding about treatment; your surgery; your radiotherapy; your chemotherapy; staying in hospital; YCS at your hospital; communication; and overall experience. Within each domain, respondents were asked whether they had received that type of treatment/service, and if so, whether particular care experiences had occurred during that phase of their treatment. They were also asked what types of professionals were involved in their care, what types of support were provided, and how satisfied they were with their care. Questions about psychosocial support focused on the support provided by the YCS, since that is one of the YCS's core functions.^18^

The U.S. version of the survey was similar to the Australian version, with small alterations to reflect systemic differences in care provision. For example, when asking about psychosocial support, the U.S. version asked about support received from both hospital/cancer services and from other supports such as online and community organizations. It contained a maximum of 125 questions.

Participants gave their survey responses on a 5-point Likert scale. For example, in response to the question “Was fertility discussed in a sensitive, supportive way?” the response options were: (1) Yes, definitely; (2) Yes, I think so; (3) No, I don't think so; (4) No, definitely not; and (5) Not sure/can't remember. Consistent with the approach used with the original AYA Experience of Care survey,^22^ responses were dichotomized into Yes (responses 1–2) or No/Unsure (responses 3–5).

Both versions of the survey used branching logic to only present questions that were relevant to the participant. The U.S. version automatically skipped all irrelevant items based on previous responses. The Australian version used automatic skipping at the domain level but also provided the response option “this wasn't needed” for some items, which was treated as a skip. The estimated time for survey completion ranges between 15 and 30 minutes if done in one continuous sitting, depending on the number of skips.

### Analysis

In accordance with each country's privacy legislation, the combined international dataset was limited to aggregated, categorical data (proportions and denominators). We compared the proportions of Australian and U.S. participants who endorsed different care experiences on the YPEoC using Pearson's chi-square tests of independence, or Fisher's exact test if one or more cells had an expected size of less than 6. A similar approach was taken for satisfaction questions, where we compared the proportions of Australian and U.S. participants who said they were satisfied/very satisfied.

We made an *a priori* selection of 37 items that appeared in both countries' surveys and that were the most relevant to the AYA cancer care priority areas.^23^ We used the Benjamini-Hochberg procedure to limit the false discovery rate to 0.05,^25^ resulting in an index *p*-value of 0.0235.

We also conducted demographic comparisons between the Australian and U.S. samples, to identify possible sources of bias. No adjustment for multiple comparisons was applied to these comparisons so we could identify and account for all demographic differences. Thus, the index *p*-value for significance was set at 0.05.

Follow-up subgroup analyses explored the possible impact of demographics on survey items that were considered particularly sensitive to between-sample differences. For example, the comparison between U.S. and Australian fertility experiences was repeated separately for males and females. Given the exploratory nature of these sensitivity analyses, and the small sample sizes, no adjustment for multiple comparisons was applied, with the index *p*-value for significance set at 0.05.

## Results

### Demographic and clinical characteristics

Table 1 presents the demographic and clinical characteristics of the 118 Australian and 72 U.S. participants and associated chi-square tests. Participants from the U.S. were significantly more likely to have been female and aged over 24 at diagnosis. Australian participants were significantly more likely to have been enrolled in school at the time of recruitment. Most participants spoke English only at home (88% of Australian and 96% of U.S. AYAs).

**Table 1.** Demographic and Clinical Characteristics of Participants from Australia and the U.S.

	Australia			U.S.			% Dif	χ^2^	*p*
	%	*n*	Denom	%	*n*	Denom			
Age at diagnosis			118			72		31.165	<0.001
15–19	32	38		21	15		11		
20–24	34	40		31	22		3		
25–29	7	8		49	35		−42		
Missing^a^	27	32		0	0				
Gender			118			72		7.901	0.005
Female	61	72		81	58		−20		
Male	39	46		19	14		+20		
Languages spoken at home			116			71		3.279	0.070
English only	88	102		96	68		−8		
Other language/s	12	14		4	3		+8		
Highest level of education			115			72		12.142	<0.001
Some high school	18	21		1	1		+17		
Completed high school+	82	94		99	71		−17		
Current occupation									
Studying	60	69	115	42	30	72	18	5.974	0.015
In paid work	60	68	114	69	50	72	−10	1.826	0.177
Cancer type			115			72		3.812	0.051
Hematological (blood)	48	55		33	24		15		
Oncological (solid tumor)	52	60		67	48		−15		
Diagnosis^b^			115			72			
Lymphoma	37	43		18	13		19		
Leukemia	10	12		15	11		−5		
Bone or soft tissue sarcoma	19	22		6	4		14		
Brain or CNS	8	9		7	5		1		
Testicular	8	9		7	5		1		
Melanoma	3	4		4	3		−1		
Ovarian	3	3		10	7		−7		
Bowel/colon	3	3		1	1		1		
Breast	2	2		14	10		−12		
Cervical	1	1		3	2		−2		
Thyroid	0	0		6	4		−6		
Other^c^	6	7		10	7		−4		
Treatment received									
Chemotherapy	82	92	112	83	59	71	−1	1.203	0.868
Surgery	68	76	112	74	53	72	−6	0.692	0.405
Radiotherapy	41	46	112	40	28	70	1	0.020	0.886
Inpatient care	78	87	112	65	46	71	13	3.636	0.057
Additional staff involved									
Nurse (cancer care coordinator)	96	107	112	50	36	72	46	52.475	<0.001
Psychologist/social worker	92	103	112	26	19	72	66	84.353	<0.001
Occupational therapist/physical therapist	43	48	112	28	20	72	15	4.277	0.039
Don't know	1	1	112	0	0	72	1		
Other^d^	50	56	112	26	19	72	24		

^a^ Exact age at diagnosis could not be calculated for some Australian cases who chose not to provide their date of birth and/or their date of diagnosis on the survey. These questions were optional for Australian participants, since their study eligibility had already been checked and confirmed by the recruiting health care professionals.

^b^ Statistical comparisons were not performed at the diagnosis level due to the large number of categories and small cell sizes.

^c^ Other includes desmoid tumor, endometrial, head and neck, kidney, lung, neuroblastoma, neuroendocrine, pseudomyxoma peritonei, rectal, and skin (non-melanoma).

^d^ Other includes clinical trial nurses, education/career coordinators, medical specialists/consultants, music therapists, peer workers, prosthetists, and radiation therapists.

CNS, central nervous system.

Participants had a mix of cancer diagnoses in the proportions expected. There was a non-significant trend suggesting that Australian participants may have been more likely to have a hematological cancer, with higher proportions of Australian participants having lymphoma and sarcoma, and a higher proportion of U.S. participants having breast cancer.

There was no evidence of a significant difference between countries in treatments received, but Australian participants were significantly more likely to report receiving care from several types of multidisciplinary service providers including nurse coordinators, psychologists or social workers, and occupational therapists or physical therapists. There was also a non-significant trend suggesting that Australian participants may have been more likely to receive inpatient care, which is consistent with the diagnostic differences noted earlier.

#### Satisfaction with cancer treatment and service

Table 2 presents the proportion of Australian and U.S. participants who were satisfied or very satisfied with their experience of medical and overall care. There was no evidence of a significant difference in satisfaction with each component of medical care, but a significantly higher proportion of Australian participants were satisfied with the overall support they received from their cancer service.

**Table 2.** Proportion of Australian and U.S. Participants Who Were Satisfied or Very Satisfied with Their Experiences of Medical and Overall Care

	Australia			U.S.			% Dif	χ^2^	*p*
	%	*n*	Denom	%	*n*	Denom			
Chemotherapy	96	76	79	86	51	59	10	4.39	0.054^a^
Surgery	93	70	75	88	46	52	5	0.92	0.356^a^
Radiotherapy	91	42	46	79	22	28	13	2.41	0.164^a^
Hospital admissions	91	79	87	85	39	46	6	1.09	0.296
Support from cancer service	96	107	111	70	50	71	26	24.65	<0.001

^a^ Analysis used Fisher's exact test because one or more cells had an expected count of less than 6.

#### Fertility preservation

Table 3 presents the proportion of Australian and U.S. participants who endorsed different fertility-related care experiences in the survey. In the first fertility question, all participants were asked if they had been told about possible impacts of treatment on their fertility. Australian participants were significantly more likely to endorse this item than U.S. participants.

**Table 3.** Proportion of Australian and U.S. Participants Endorsing Different Fertility-Related Experiences

	Australia			U.S.			% Dif	χ^2^	*p*
	%	*n*	Denom	%	*n*	Denom			
Told about possible impacts on fertility									
Combined sample	90	101	112	72	52	72	18	10.09	0.001
Females only	89	62	70	69	40	58	20	7.52	0.006
Males only^a^	93	39	42	100	12	12	−7		
<25 years at diagnosis	92	70	76	77	25	37	25	11.19	0.001
Discussed in a sensitive way^b^									
Combined sample	92	87	95	71	37	52	20	10.62	0.001
Females only	91	51	56	68	27	40	24	8.51	0.004
Males only	92	36	39	83	10	12	9	0.84	0.580^b^
<25 years at diagnosis	92	60	65	67	19	25	16	4.48	0.066^b^
Told about preservation options^b^									
Combined sample	95	90	95	75	39	52	20	12.18	<0.001
Females only	93	52	56	70	28	40	23	8.78	0.003
Males only	97	38	39	92	11	12	6	0.81	0.419^b^
<25 years at diagnosis	95	62	65	81	17	25	27	12.62	0.001^b^
Referred to fertility specialist^b^									
Combined sample	87	77	89	56	29	52	31	16.63	<0.001
Females only	89	47	53	55	22	40	34	13.51	<0.001
Males only	83	30	36	58	7	12	25	3.18	0.113^b^
<25 years at diagnosis	88	53	60	59	13	25	36	13.42	<0.001

^a^ No statistical comparison could be performed, because one cell was 0.

^b^ Participants were only asked this question if they said they had been told about the possible impacts of their cancer/treatment on fertility. Participants who said this question was not relevant to them were also excluded from the analysis.

^c^ Analysis used Fisher's exact test, because one or more cells had an expected count of less than 6.

Follow-up analyses showed that this effect was only significant for females. As can be seen in Table 3, similar proportions of females (89%) and males (93%) were told about the possible impacts in Australia, whereas in the United States this information was given to 100% of males but only 69% of females. When the analysis was limited to participants aged under 25 years, the effect remained significant.

The remaining three fertility items were only answered by participants who said they had been told about the possible impacts of treatment on their fertility. Once again, Australian participants were significantly more likely to endorse each fertility experience item, with follow-up analyses showing that each effect was significant for females, but not males. When the analysis was limited to participants aged under 25 years, the effects remained significant, except for one item that asked whether fertility had been discussed in a sensitive way.

#### Age-appropriate communication

Table 4 presents the proportion of Australian and U.S. participants who endorsed experiences relating to communication with health professionals. Australian participants were significantly more likely to endorse thirteen of the communication experiences examined, including: understanding the information provided at diagnosis; being happy with their involvement in decision-making; receiving information relevant to their age group; and feeling they were treated like an AYA. U.S. participants were more likely to have been the only person involved in making treatment decisions.

**Table 4.** Proportion of Australian and U.S. Participants Endorsing Different Communication Experiences

	Australia			U.S.			% Dif	χ^2^	*p*
	%	*n*	Denom	%	*n*	Denom			
Diagnosis and decision making									
Understood information	81	91	113	61	44	72	19	15.97	0.004
Satisfied/very satisfied with diagnosis communication	89	101	113	65	47	72	24	25.96	<0.001
Was only person involved in treatment decisions									
All participants	9	10	111	29	21	72	−20	12.61	<0.001
<25 years at diagnosis	8	6	75	31	11	35	−23	10.03	0.002
Happy with involvement in decisions									
All participants	95	105	111	82	59	72	13	7.51	0.006
<25 years at diagnosis	96	73	76	80	28	35	16	7.53	0.022^a^
Information provision									
Relevant to age									
All participants	84	93	111	38	27	71	46	40.36	<0.001
<25 years at diagnosis	84	64	76	40	14	35	44	22.42	<0.001
AYA services	96	107	112	76	38	50	20	14.05	<0.001
Things to do/not do during treatment (e.g., drugs/alcohol)	93	101	109	65	46	71	28	22.31	<0.001
Why medications were important	96	97	101	80	57	71	16	11.05	0.001
Long-term effects of treatment	95	104	110	58	41	71	37	36.67	<0.001
Looking after physical health	94	104	111	42	30	71	51	59.01	<0.001
Communication and engagement									
Treated like an AYA	89	98	110	72	51	71	17	8.83	0.003
Loved one present when given important information	86	96	111	93	66	71	−6	1.85	0.173
Opportunity to speak to HCPs alone	94	104	111	83	59	71	11	5.20	0.023
Family/loved ones had opportunity to talk to HCPs	83	92	111	77	55	71	5	0.82	0.366
Could trust HCPs with personal information	96	107	111	92	65	71	5	1.96	0.162
HCPs knew enough about young people and cancer	95	104	110	56	40	71	38	38.73	<0.001
Satisfied/very satisfied with communication	98	109	111	79	56	71	19	19.10	<0.001

^a^ Analysis used Fisher's exact test, because one or more cells had an expected count of less than 6.

AYA, adolescent and young adult.

There was no evidence of a significant difference in the proportion of respondents who felt that their family or loved ones had enough opportunities to talk to the health professionals, who felt they could trust their health professionals with important information, or who reported having a loved one present when they were given important information.

Follow-up chi-square tests with participants aged under 25 were conducted for three items: being the only one involved in making decisions about treatment; being happy with their involvement in decision making; and being given information relevant to their age group. In each case, the difference between countries was still significant.

#### Direct provision of psychosocial support

Table 5 presents the proportion of Australian and U.S. participants endorsing different experiences of psychosocial support. U.S. participants were significantly more likely to report that they needed support that was not provided by their cancer care team (this item was endorsed by 4% of Australian AYAs and 41% of U.S. AYAs). U.S. participants were also significantly more likely to have been offered the chance to meet with other young cancer patients or survivors. For all other psychosocial support items analyzed, Australian participants were significantly more likely to endorse the experience described. This included: being asked questions to see what support was needed; being offered emotional support; their loved ones being offered support; and receiving ongoing support.

**Table 5.** Proportion of Australian and U.S. Participants Endorsing Different Experiences of Psychosocial Support

	Australia			U.S.			% Dif	χ^2^	*p*
	%	*n*	Denom	%	*n*	Denom			
Asked questions regarding coping and support needs	90	101	112	72	31	43	18	8.042	0.005
Offered a chance to meet other AYA patients/survivors	73	81	111	91	39	43	−18	5.660	0.017
Offered to help discuss cancer with family/friends	82	91	111	40	17	43	42	26.658	<0.001
Offered help with returning to education/work	86	95	111	28	12	43	58	48.625	<0.001
Offered financial help/advice	83	92	111	26	11	42	57	44.515	<0.001
Offered help with completing forms	82	92	112	16	7	43	66	58.410	<0.001
Offered any emotional support	98	106	108	49	35	72	50	62.460	<0.001
Received ongoing support	97	109	112	49	35	71	48	59.765	<0.001
Needed other support not provided	4	5	112	41	29	71	−36	38.019	<0.001
Family/loved ones offered any support	83	93	112	25	18	71	58	60.587	<0.001
Loved ones told about other support organizations	80	90	112	20	14	71	61	65.128	<0.001

## Discussion

This study offers important insights into the way that AYAs experience cancer care in two countries with different approaches to service coordination, funding, and accountability. The results suggest that while most patients in both Australia and the U.S. are satisfied with their medical treatment, there is a significant difference in how AYAs rate the support they receive from their cancer service. Australian young people reported higher satisfaction levels than U.S. participants in their overall support, along with better experiences of care in relation to fertility support, age-appropriate communication, and most notably, psychosocial support.

In seeking to understand and explain these differences, the presence or absence of government funding and national coordination of clinical services appear to be important contributing factors.

As noted earlier, there is both state and federal funding and national oversight of AYA cancer care in Australia, with a single Youth Cancer Framework to guide operations nationally in the provision of best practice coordinated multidisciplinary care.^17^ This strategy improves consistency in service delivery and promotes sharing of expertise, ideas, and learnings between services.^13,15^ Consistent with this, a study in the United Kingdom found that AYA patients were more likely to report unmet information and support needs when treated outside specialist AYA cancer treatment centers, and recommended that health care professionals seeing AYA patients elsewhere should be provided with support from the AYA cancer treatment centers.^26^

In the United States, there is no national framework to standardize care delivery between hospitals and there is also more variation in funding sources between services. Access to care is based on insurance coverage and individual financial resources, with the most common payors for AYA cancer treatment being employers or university insurance programs.^27^ While most U.S. participants in our study were insured at the time of diagnosis,^24^ Parson et al. found that 25% of U.S. AYAs reported some period without insurance coverage during the 3 years post-diagnosis.^27^

This international variation in funding approaches could potentially help to explain the different experiences of fertility preservation we identified in our study. Access to fertility preservation is particularly inconsistent in the United States, and even when insured, many U.S. patients have out-of-pocket costs. Our survey found that Australian participants were significantly more likely to have been told about possible impacts on fertility.

Further, of those who had been told about the possible impacts, Australian participants were more likely to report that fertility was discussed in a sensitive way, to have been told about preservation options, and to have received a fertility referral. In the United States, provider concern about patients' ability to pay for fertility preservation has previously been shown to be a barrier to these discussions.^28^

To account for the gender differences between our Australian and U.S. samples, and since fertility preservation is often simpler and cheaper for males,^29^ we compared Australian and U.S. responses with our fertility questions for females and males separately in a follow-up analysis. This revealed that the differences between countries only applied to female participants.

It is possible that U.S. doctors are less likely than Australian doctors to talk to their female patients about fertility preservation options or to provide them with fertility referrals, because they are too expensive or difficult to access.^30^ However, such constraints should not preclude providers in all countries from informing patients in a sensitive way about their risk to fertility, consistent with the American Society of Clinical Oncology (ASCO) and other guidelines.^31^

In relation to age-appropriate communication, U.S. AYAs were significantly more likely to have endorsed being the only decision maker from their family present when discussing their cancer treatment regimen with their treatment team. This means that for Australian AYAs, treatment decision making was more likely to be a joint process involving more than one family member. Australian AYAs were also more likely to report receiving age-appropriate information.

To account for age differences between the samples, these analyses were repeated for participants under 26 years of age, and the findings were consistent. Concerningly, U.S. AYAs were significantly less likely to say they understood the information provided at diagnosis, to feel that they were treated like an AYA, or to believe that their health care professionals knew enough about young people and cancer.

The high proportions of Australian participants endorsing positive communication experiences suggest that Australia may have more consistent levels of age-appropriate care between sites. This may be due in part to Australia's national approach to AYA cancer care, with a higher degree of variability arising from a system without national guidelines or coordination (the U.S.).

Perhaps the most compelling difference in experiences of care relates to psychosocial support, with Australian participants significantly more likely to have been offered and/or received support across a wide range of domains. Australian AYAs were also more likely to report that they had a nurse in the role of cancer care coordinator and were more likely to have had psychologists, social workers, occupational therapists, and physical therapists involved in their cancer care.

We have previously reported that U.S. AYAs are more likely to receive psychosocial support from online and community organizations, with less than half being provided with information about AYA support services by their health care team.^24^ This suggests that many U.S. AYAs may be required to source their own support services. In contrast, Australian AYAs generally receive psychosocial support directly from their YCS as a routine part of treatment.^13^

Given this, it may be unsurprising that U.S. AYAs were more likely to miss out on certain critical age-specific interventions such as those related to their social support network, education, or work. Our finding that ∼40% of U.S. AYAs reported unmet needs for support is congruent with previous reports from the United States.^32^ However, it should be noted that U.S. AYAs were more likely than Australian AYAs to have been offered the chance to meet other AYAs with cancer, which often involves connecting with community organizations outside the hospital.

Study limitations include that on average, U.S. participants were significantly older and more likely to be female than Australian participants, with non-significant trends suggesting between-country differences in relation to cancer type and inpatient care. All of these may have biased the results. While the nature of the data did not allow us to control for these variables in the main analyses, we endeavored to account for the age and gender differences by conducting relevant follow-up subgroup analyses.

The different recruitment methods used between countries may have resulted in other forms of selection bias that we were unable to account for. For both countries, it is also possible that self-selection played a role such that AYAs who felt more satisfied or engaged with their health care may have been more likely to participate.

Further, the low proportion of participants who spoke a language other than English at home suggests that both samples were not representative of the ethnic/linguistic diversity of their respective populations, which would limit the generalizability of our conclusions. Finally, the high rates of insurance coverage of our U.S. participants^24^ may have influenced our findings, with the experiences of those without insurance potentially comparing even less favorably to the experiences of Australian AYAs.

Future research could compare Australian and U.S. experiences of care with those of AYA patients from the United Kingdom (whose system is relatively similar to Australia) and from other countries with different models of care. It would be useful to make the survey available in different languages, and to control for the possible impact of treating hospital type (pediatric, adult, and/or specialist AYA service; community or academic center).^22,24^

It should also be acknowledged that gaps remain in our understanding about how and whether the provision of specialist AYA care translates to improvements in other patient-reported outcomes, such as quality of life.^33^ Future research could compare countries' performance in relation to both PREMs and an AYA-specific measure of quality of life, and examine the relationship between these outcomes.

Despite these limitations, this study is notable for permitting a comparison of the cancer care experiences of young people between countries. To our knowledge, this has not been done before. Experience of care measures provide a useful paradigm for such comparisons, since they are grounded in internationally applicable best practice guidelines for care while allowing AYAs' voices to be heard in relation to their individual experiences.

## Conclusion

The unique needs of AYA cancer patients demand consistent, age-appropriate best practices to achieve optimal medical and psychosocial outcomes. This study demonstrates the potential benefits of a nationally coordinated, evidence-based, specialized system of support services for AYAs with cancer, allowing hospitals to deliver high value care without needing to develop their own system for patient management. In addition, such standardization allows AYA cancer care providers to receive specialized training and communicate with professionals in similar roles throughout the country.

## References

[1] Bleyer A, Ferrari A, Whelan J, et al. Global assessment of cancer incidence and survival in adolescents and young adults. Pediatr Blood Cancer 2017;64(9); doi: 10.1002/pbc.26497 28244694

[2] Fidler MM, Gupta S, Soerjomataram I, et al. Cancer incidence and mortality among young adults aged 20–39 years worldwide in 2012: A population-based study. Lancet Oncol 2017;18(12):1579–1589. 29111259 10.1016/S1470-2045(17)30677-0

[3] Miller KD, Fidler-Benaoudia M, Keegan TH, et al. Cancer statistics for adolescents and young adults, 2020. CA Cancer J Clin 2020;70(6):443–459. 32940362 10.3322/caac.21637

[4] AIoHa W. Cancer in Adolescents and Young Adults in Australia. AIHW: Canberra; 2018.

[5] Prasad PK, Hardy KK, Zhang N, et al. Psychosocial and neurocognitive outcomes in adult survivors of adolescent and early young adult cancer: A report from the childhood cancer survivor study. J Clin Oncol 2015;33(23):2545–2552; doi: 10.1200/JCO.2014.57.7528 26150441 PMC4525049

[6] Suh E, Stratton KL, Leisenring WM, et al. Late mortality and chronic health conditions in long-term survivors of early-adolescent and young adult cancers: A retrospective cohort analysis from the Childhood Cancer Survivor Study. Lancet Oncol 2020;21(3):421–435; doi: 10.1016/s1470-2045(19)30800-9 32066543 PMC7392388

[7] Osborn M, Little C, Bowering S, et al. Youth Cancer Services in Australia: Development and implementation. International perspectives on AYAO, part 3. J Adolesc Young Adult Oncol 2013;2(3):118–124; doi: 10.1089/jayao.2012.0032 26812190

[8] Fardell JE, Patterson P, Wakefield CE, et al. A narrative review of models of care for adolescents and young adults with cancer: Barriers and recommendations. J Adolesc Young Adult Oncol 2018;7(2):148–152; doi: 10.1089/jayao.2017.0100 29298105

[9] Zeltzer LK. Cancer in adolescents and young adults psychosocial aspects. Long-term survivors. Cancer 1993;71(S10):3463–3468. 8490896 10.1002/1097-0142(19930515)71:10+<3463::aid-cncr2820711753>3.0.co;2-b

[10] Patterson P, D'Agostino NM, McDonald FEJ, et al. Screening for distress and needs: Findings from a multinational validation of the Adolescent and Young Adult Psycho-Oncology screening tool with newly diagnosed patients. Psychooncology 2021 30(11):1849–1858; doi: 10.1002/pon.5757 34160847 PMC9291177

[11] Patterson P, McDonald FE, Zebrack B, et al. Emerging issues among adolescent and young adult cancer survivors. Semin Oncol Nurs 2015;31(1):53–59; doi: 10.1016/j.soncn.2014.11.006 25636395

[12] Barnett M, McDonnell G, DeRosa A, et al. Psychosocial outcomes and interventions among cancer survivors diagnosed during adolescence and young adulthood (AYA): A systematic review. J Cancer Surviv 2016;10(5):814–831; doi: 10.1007/s11764-016-0527-6 26920873 PMC5001943

[13] Patterson P, Allison KR, Bibby H, et al. The Australian Youth Cancer Service: Developing and monitoring the activity of nationally coordinated adolescent and young adult cancer care. Cancers 2021;13(11):2675; doi: 10.3390/cancers13112675 34071622 PMC8198716

[14] Johnson RH. AYA in the USA. International perspectives on AYAO, part 5. J Adolesc Young Adult Oncol 2013;2(4):167–174. 24380035 10.1089/jayao.2012.0027 PMC3869433

[15] Osborn M, Johnson R, Thompson K, et al. Models of care for adolescent and young adult cancer programs. Pediatr Blood Cancer 2019;66(12):e27991; doi: 10.1002/pbc.27991 31524328

[16] CanTeen Australia. Youth Cancer Services Activity Data 2015. (unpublished dataset); Canteen: Sydney, Australia; 2015.

[17] Canteen Australia. The Australian Youth Cancer Framework for Adolescents and Young Adults with Cancer. Canteen Australia: Sydney, Australia; 2017.

[18] Cancer Australia, Canteen. National service delivery framework for adolescents and young adults with cancer. Commonwealth of Australia: Canberra; 2008.

[19] Bleyer A. Young adult oncology: The patients and their survival challenges. CA Cancer J Clin 2007;57(4):242–255; doi: 10.3322/canjclin.57.4.242 17626120

[20] Doyle C, Lennox L, Bell D. A systematic review of evidence on the links between patient experience and clinical safety and effectiveness. BMJ Open 2013;3(1):e001570; doi: 10.1136/bmjopen-2012-001570 PMC3549241 23293244

[21] Bibby H, White V, Thompson K, et al. What are the unmet needs and care experiences of adolescents and young adults with cancer? A systematic review. J Adolesc Young Adult Oncol 2017;6(1):6–30; doi: 10.1089/jayao.2016.0012 27454408

[22] White V, Skaczkowski G, Thompson K, et al. Experiences of care of adolescents and young adults with cancer in Australia. J Adolesc Young Adult Oncol 2018;7(3):315–325; doi: 10.1089/jayao.2017.0104 29356588

[23] Bleyer A, Barr R. Cancer in young adults 20 to 39 years of age: Overview. Semin Oncol 2009;36:194–206. 19460577 10.1053/j.seminoncol.2009.03.003

[24] Jacobsen RL, Macpherson CF, Pflugeisen BM, et al. Care experience, by site of care, for adolescents and young adults with cancer. JCO Oncol Pract 2021;17(6):e817–e826; doi: 10.1200/OP.20.00840 33566700

[25] Benjamini Y, Hochberg Y. Controlling the false discovery rate: A practical and powerful approach to multiple testing. J R Stat Soc Series B Stat Methodol 1995;57(1):289–300.

[26] Martins A, Alvarez-Galvez J, Fern LA, et al. The BRIGHTLIGHT National Survey of the impact of specialist teenage and young adult cancer care on caregivers' information and support needs. Cancer Nurs 2021;44(3):235–243. 31895170 10.1097/NCC.0000000000000771

[27] Parsons HM, Schmidt S, Harlan LC, et al. Young and uninsured: Insurance patterns of recently diagnosed adolescent and young adult cancer survivors in the AYA HOPE study. Cancer 2014;120(15):2352–2360; doi: 10.1002/cncr.28685 24899580 PMC4108530

[28] Logan S, Perz J, Ussher J, et al. Clinician provision of oncofertility support in cancer patients of a reproductive age: A systematic review. Psychooncology 2018;27(3):748–756; doi: 10.1002/pon.4518 28762627

[29] Gunasheela D, Gunasheela S. Strategies for fertility preservation in young patients with cancer: A comprehensive approach. Indian J Surg Oncol 2014;5(1):17–29. 24669162 10.1007/s13193-014-0291-x PMC3964233

[30] Letourneau JM, Smith JF, Ebbel EE, et al. Racial, socioeconomic, and demographic disparities in access to fertility preservation in young women diagnosed with cancer. Cancer 2012;118(18):4579–4588; doi: 10.1002/cncr.26649 22451228 PMC3387319

[31] Oktay K, Harvey BE, Partridge AH, et al. Fertility preservation in patients with cancer: ASCO clinical practice guideline update. J Clin Oncol 2018;36(19):1994–2001. 29620997 10.1200/JCO.2018.78.1914

[32] Zebrack B, Isaacson S. Psychosocial care of adolescent and young adult patients with cancer and survivors. J Clin Oncol 2012;30(11):1221–1226; doi: 10.1200/JCO.2011.39.5467 22412147

[33] Taylor RM, Fern LA, Barber J, et al. Longitudinal cohort study of the impact of specialist cancer services for teenagers and young adults on quality of life: Outcomes from the BRIGHTLIGHT study. BMJ Open 2020;10(11):e038471; doi: 10.1136/bmjopen-2020-038471 PMC7692812 33243793

